# Trait Impulsivity Associated With Altered Resting-State Functional Connectivity Within the Somatomotor Network

**DOI:** 10.3389/fnbeh.2020.00111

**Published:** 2020-06-24

**Authors:** Aleksandra M. Herman, Hugo D. Critchley, Theodora Duka

**Affiliations:** ^1^Department of Psychology, Royal Holloway, University of London, Egham, United Kingdom; ^2^Behavioural and Clinical Neuroscience, University of Sussex, Brighton, United Kingdom; ^3^Brighton and Sussex Medical School, Brighton, United Kingdom; ^4^Sackler Centre for Consciousness Science, University of Sussex, Brighton, United Kingdom; ^5^Sussex Addiction Research and Intervention Centre, Brighton, United Kingdom

**Keywords:** trait impulsivity, resting state, functional connectivity, Barratt Impulsiveness Scale, somatomotor network

## Abstract

Knowledge of brain mechanisms underlying self-regulation can provide valuable insights into how people regulate their thoughts, behaviors, and emotional states, and what happens when such regulation fails. Self-regulation is supported by coordinated interactions of brain systems. Hence, behavioral dysregulation, and its expression as impulsivity, can be usefully characterized using functional connectivity methodologies applied to resting brain networks. The current study tested whether individual differences in trait impulsivity are reflected in the functional architecture within and between resting-state brain networks. Thirty healthy individuals completed a self-report measure of trait impulsivity and underwent resting-state functional magnetic resonance imaging. Using Probabilistic Independent Components Analysis in FSL MELODIC, we identified across participants 10 networks of regions (resting-state networks) with temporally correlated time courses. We then explored how individual expression of these spatial networks covaried with trait impulsivity. Across participants, we observed that greater self-reported impulsivity was associated with decreased connectivity of the right lateral occipital cortex (peak mm 46/-70/16, FWE 1-*p* = 0.981) with the somatomotor network. No supratheshold differences were observed in between-network connectivity. Our findings implicate the somatomotor network, and its interaction with sensory cortices, in the control of (self-reported) impulsivity. The observed “decoupling” may compromise effective integration of early perceptual information (from visual and somatosensory cortices) with behavioral control programs, potentially resulting in negative consequences.

## Introduction

Self-control allows people to make plans for the future, choose the best option from several alternatives, control impulses, inhibit unwanted thoughts, and regulate behaviors and emotions (Kelley et al., 2015). Past studies typically employed task-related functional magnetic resonance imaging (fMRI) to understand the neural substrates of transient fluctuations in self-control in different circumstances or in distinct populations. Although this approach is well-suited to capture momentary changes in brain activity in response to specific (internal or external) stimuli, it is arguably insufficient to capture more tonic aspects of self-control (Kelley et al., 2015). A global whole-brain network approach can provide more comprehensive insight into neural substrates supporting individual differences in the capacity for self-control over longer timescales. Moreover, measurement of functional connectivity (FC) across “resting-state” (RS) networks has proven value as a tool for characterizing mechanisms underlying neurocognitive processes and psychiatric disorders, while overcoming technical and inferential limitations of task-related fMRI (De Luca et al., 2006; van den Heuvel and Hulshoff Pol, 2010; Cole et al., 2014; Dipasquale et al., 2015).

Specific studies using FC at rest have tested for differences in the interaction between brain regions that account for impulsivity and, more generally, the executive function and dysfunction, in children (Inuggi et al., 2014) and in young adults (Davis et al., 2013; Reineberg et al., 2015). In typically developing children (8–12 years old) parental ratings of trait impulsivity are related to lower RS brain connectivity within the default mode network (DMN), specifically between posterior cingulate cortex and right angular gyrus (Inuggi et al., 2014). The DMN is considered a “task-negative” network, where activity is strongest when an individual is not engaged in an external task (e.g., at rest). Correspondingly, DMN activity is typically anti-correlated to other “task-related” resting-state networks (RSN) (Uddin et al., 2009). In highly impulsive children, the canonical anti-phasic relation between the DMN and action-related networks is much reduced, indicating that trait impulsivity is linked to a reduced functional segregation of task-negative and task-positive networks (i.e., the natural degree of anti-correlation between these networks is reduced). By extension, impulsivity may putatively arise in the context of functional interference between brain systems directing internal and external attention (Inuggi et al., 2014).

In adults, self-report questionnaires are used to assess trait impulsivity, measuring one’s tendencies to show premature, unplanned and short-sighted actions and decisions in daily life (Herman et al., 2018). Applying graph-theory approaches to functional brain architecture at rest in adults revealed an association between trait impulsivity and increased segregation between cortical and sub-cortical regions (i.e., increased “modularity”) (Davis et al., 2013). This is coherent with findings in young adults for whom core aspects of executive function (quantified using three behavioral tasks) were positively associated with connectivity between the frontal pole and an “attentional” RSN, and also between the cerebellum and a right frontoparietal RSN. This suggests that individuals with better executive functioning manifest more expanded yet more integrated RSN relative to individuals with worse executive functioning (Reineberg et al., 2015). However, there are also contrasting findings: Individuals with increased motor impulsivity (i.e., poorer inhibitory capacity on the go/no-go task) and higher trait impulsivity (Barratt Impulsiveness Scale), reportedly show greater RS FC between the basal ganglia and thalamus, motor cortex, temporal lobe and prefrontal cortex (Korponay et al., 2017). This suggests that increased connectivity between motor-brain regions may predispose to disinhibited actions.

The comparison between these earlier studies to disentangle the observed differences, however, is hindered by the different approaches to functional connectivity (e.g., focus on a single, pre-defined RSN, or the use of seed-based methods, instead of a whole-brain model-free approach) and diverse measures of disinhibited behaviors used (various behavioral tasks or trait measures). Their focus on general executive functioning instead of trait impulsivity or a specific behavioral impulsivity task is also a limitation. The present study set out to cover these gaps by testing for predicted associations between trait impulsivity and the strength of FC within as well as between resting-state networks.

An understanding of the brain mechanisms underlying self-regulation can provide valuable insights into how people regulate and control their thoughts, behaviors, and emotional states and can illuminate what happens on those occasions when this regulation fails (Kelley et al., 2015). The present study tested whether individual differences in trait impulsivity are reflected in within-and between-resting-state network architecture using a FC approach. Based on previous findings, we predicted that internal architecture of the default mode (Inuggi et al., 2014), frontoparietal, and attentional networks (Reineberg et al., 2015) would be linked to the expression impulsivity across individuals and that between-network connectivity pattern of task-negative (DMN) and task-positive networks (Inuggi et al., 2014) might also be modulated by the magnitude of trait impulsivity.

## Materials and Methods

### Participants

Thirty volunteers (nine males) were recruited from staff and students of the University of Sussex. Participants were required to be between 18 and 40 years old and right-handed. Exclusion criteria included history of any psychological or neurological disorders, head injury, current treatment for any psychological or physical condition (including use of inhalers; excluding the contraceptive pill), pregnancy or breastfeeding, clinically significant impairment of vision, use of psychoactive substances 48 h before testing, and any MRI contradictions (claustrophobia, having any metal implants, teeth braces or bridges, or cardiac pacemakers).

All participants provided written informed consent. The study was conducted according to the Declaration of Helsinki. All procedures were approved by the Brighton and Sussex Medical School Research Governance and Ethics Committee.

### Questionnaires

Participants completed the *Barratt Impulsiveness Scale* (BIS; Patton et al., 1995), a 30-item questionnaire with three distinct impulsivity facets: attentional (eight items; a lack of focus on the ongoing task), motor (11 items; acting without thinking), and non-planning impulsivity (11 items; orientation to the present rather than to the future).

### MRI Experiment Design

In the MRI scanner, first, a structural scan was obtained followed by a 7-min resting-state scan (165 volumes) during which participants were instructed to rest with their eyes open focusing on a fixation cross in the center of the screen with the instruction to try not to think of anything and not to fall asleep. All participants were tested between 2 pm and 6 pm to control for possible time of day effects on an attentional level.

#### MRI Acquisition

MRI was performed on a 1.5-Tesla MAGNETOM Avanto scanner (Siemens AG, Munich, Germany) with upgraded gradients and a 32-channel headcoil. Structural volumes were obtained using a high-resolution three-dimensional magnetization prepared rapid acquisition gradient echo sequence. Functional data sets used T2^∗^-weighted echo planar imaging sensitive to blood oxygenation–level-dependent signal (repetition time = 2.52 s, echo time = 43 ms, flip angle = 90°, 34 slices, 3-mm slice thickness, field of view = 192 mm, voxel size = 3 × 3 × 3 mm). Slices were angled −30° in the anteroposterior axis to reduce the signal loss in orbitofrontal regions (Deichmann et al., 2003; Weiskopf et al., 2006).

#### fMRI Data Pre-processing

Imaging analysis was performed using FEAT (FMRI Expert Analysis Tool) version 6.00, a part of FMRIB Software Library (FSLv6.0, Jenkinson et al., 2012). Pre-processing steps included (1) skull stripping of structural images with Brain Extraction Tool (BET), (2) removal of the first four functional volumes to allow for signal equilibration, (3) head movement correction by volume-realignment to the middle volume using MCFLIRT, (4) global 4D mean intensity normalization, (5) spatial smoothing (6mm full-width half-maximum), and (6) noise signals removal, (7) temporal high-pass filtering (100 s cut-off).

FMRI datasets were co-registered to the participant’s structural image using affine boundary-based registration as implemented in FSL FLIRT (Jenkinson and Smith, 2001; Jenkinson et al., 2002) and subsequently transformed them to MNI152 standard space with 2 mm isotropic resolution using non-linear registration through FSL FNIRT (Andersson et al., 2010). Noise signals were identified individually and removed using ICA-AROMA toolbox (Pruim et al., 2015). ICA-AROMA incorporates probabilistic Independent Component Analysis (ICA) on the partly pre-processed single-subject fMRI data (following spatial smoothing and normalization but before high-pass filtering), identifies independent components (ICs) representing motion artifacts and removes them from the fMRI time-series using linear regression.

Since there was a broad age range within our population (18–37 years) and a larger number of females than males participated in the study, in all reported analyses, gender and mean-centered age were added as covariates of no interest.

#### Independent Components Analysis

**FIGURE 1:**
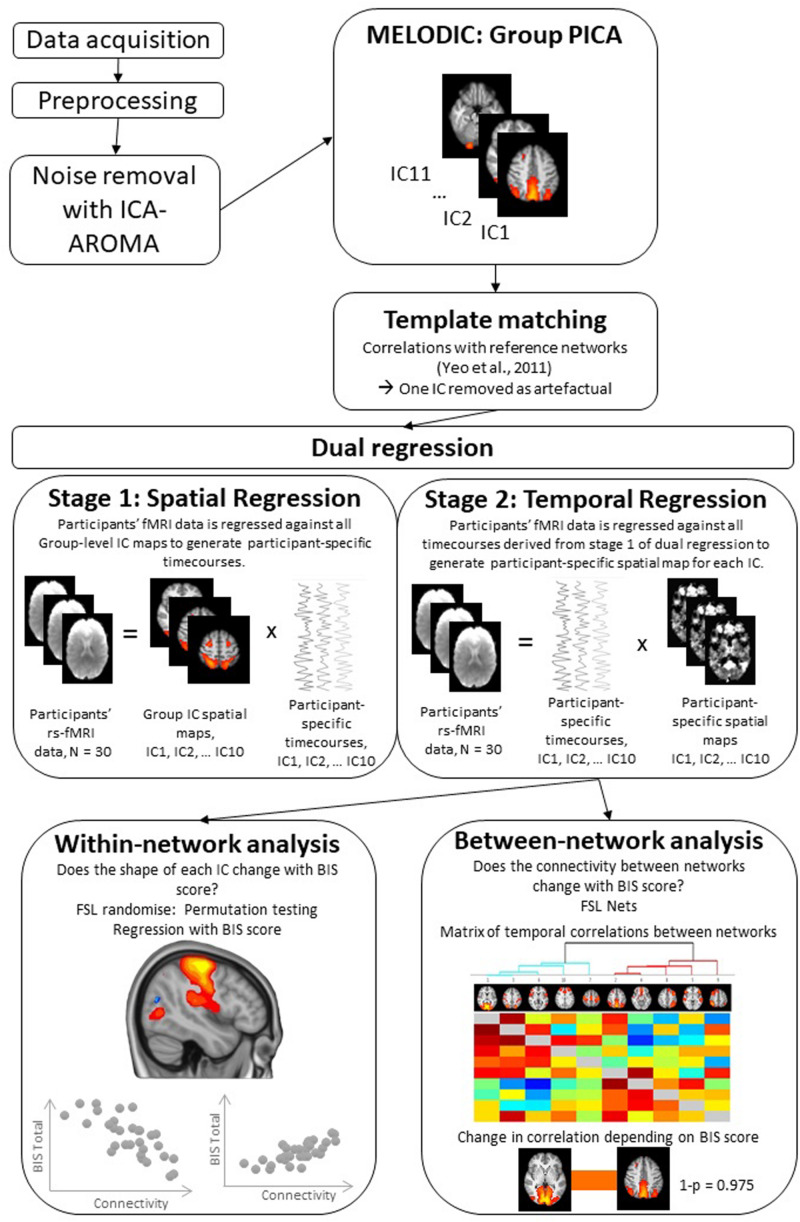
Illustration of the steps followed during resting-state functional fMRI data analysis. For more details of this analysis steps see Methods section. rs-fMRI, Resting state functional magnetic resonance imaging; PICA, Probabilistic Independent Component Analysis; IC, Independent Component; BIS, Barratt Impulsiveness Scale.

The RS data analysis pipeline is summarized in Figure 1. To decompose the RS data into various independent spatiotemporal components, Probabilistic Independent Components Analysis (PICA) was performed on the pre-processed functional scans using Melodic version 3.14 (Beckmann and Smith, 2004). A dimensionality estimation using the Laplace approximation to the Bayesian evidence of the model order (Beckmann and Smith, 2004) produced 11 spatiotemporal components. Following an approach described in Reineberg et al. (2015), we statistically compared the spatial map of each independent component (IC) to a set of seven reference RS networks from a previous large-scale RS analysis (Yeo et al., 2011). We used FSL’s “fslcc” tool to calculate Pearson’s r for each pairwise relationship and kept only those ICs that yielded a significant spatial correlation (Pearson’s *r* > 0.3) with one of the reference networks. This procedure identified and helped label 10 target ICs (see Table 1 for details). Upon visual inspection, the remaining 1 IC was considered noise and was not subjected to further analysis.

**TABLE 1:** Identified Independent Components (IC Number) and their characteristics.

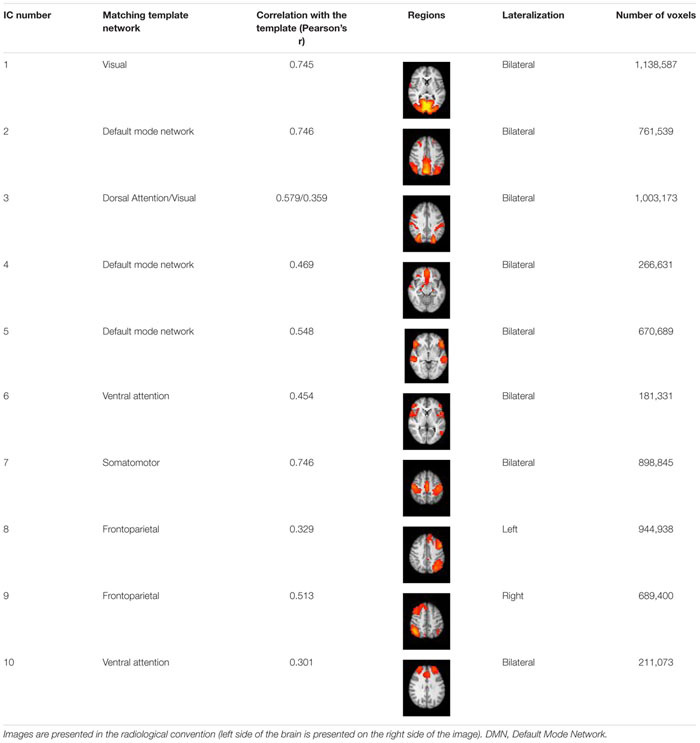

##### Dual regression

For the between-subject analysis, we carried out dual regression, a technique that back-reconstructs each un-thresholded group-level component map at the individual subject level, generating participant-specific spatial maps and time courses (Beckmann et al., 2009; Filippini et al., 2009). The dual regression consists of (1) a spatial regression of the group-average set of ICs that produces a set of participant-specific time series, one per group-level component, and (2) a temporal regression of those participant specific time series, resulting in a set of participant-specific spatial maps, one per group-level component (see Figure 1). Participant-specific components are whole brain images. For some individuals, the given IC might be very similar to the group level IC while others might show variations of the group level IC (i.e., have an expanded/constrained network or high/low connectivity of a particular region). Statistical analyses (discussed below) are performed on these whole brain participant-specific ICs to determine areas that covary with trait impulsivity measure, that is BIS total score.

##### Within-network connectivity

To quantify the within-network variation in functional connectivity (FC), depending on BIS total score and participant-specific ICs, we carried out voxel-wise regression to assess statistically significant differences in FC in relation to trait impulsivity score. The analysis was conducted using Randomize, FSL’s non-parametric permutation testing tool (Winkler et al., 2014), with 5000 permutations and threshold-free cluster enhancement (TFCE) with an alpha level of 0.05 to correct for multiple comparisons. The permutation testing procedure was run for each set of participant-specific ICs (one for each group-level ICs of interest); thus, the resulting statistical images reveal how variation in RS FC (functional connectivity estimates) predict differences in trait impulsivity (see Figure 1). For example, the permutation testing procedure could reveal that individuals with expanded one of the ICs (i.e., expanded to areas outside the areas included in the group-level IC) report greater impulsivity. Following studies using similar procedures (Uddin et al., 2013; Nomi and Uddin, 2015; Reineberg et al., 2015; de Bézenac et al., 2017; Herman et al., 2019), further correction for multiple component testing was not applied.

##### Between-network connectivity

*FSLNets*. To examine the relationship between trait impulsivity and between-network FC, we employed the FSLNets package^1^ implemented in Matlab v2015b (The MathWorks, 2015). This analysis involved correlation of the participants’ time courses from the dual regression analysis and subjected them to between-network comparisons to determine how they are correlated with each other (Smith et al., 2013). We then calculated full and partial correlations between all pairs of ICs. Partial correlations are computed as correlations between two ICs while controlling for the effect of all other ICs and are thought to reflect more direct connections (Smith et al., 2011). Finally, BIS total score was used as a regressor in the regression analysis in FSL randomize with 5000 permutations to assess differences in between-network connectivity across BIS spectrum. Results were FWE corrected for multiple comparisons.

## Results

### Participants

No participant was removed because of extensive motion in the scanner. The final sample (*N* = 30, 9 males) was aged between 18 and 37 years old (*M* = 23.40, *SD* = 5.01). The average BIS Total score was 65.30 ± 11.39.

### Within-Network Connectivity

**FIGURE 2:**
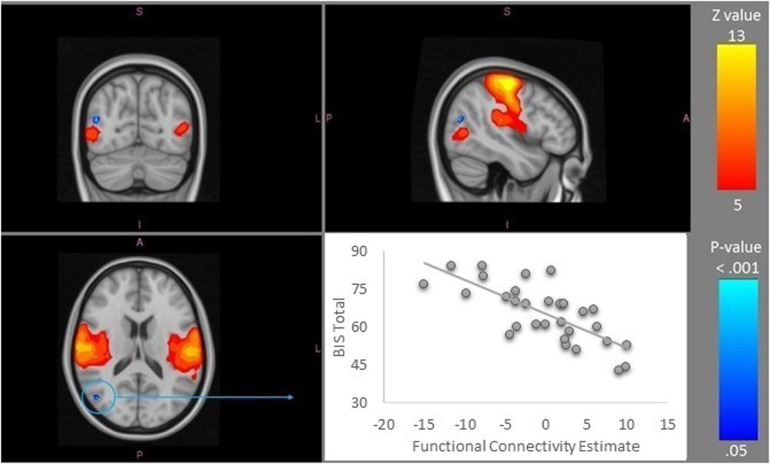
Somatomotor resting-state network (IC 7), depicted in warm colors, was identified as the only network showing the significant differences across the BIS total score spectrum. The area that showed reduced resting state functional connectivity within this network was a region in the lateral occipital cortex (in blue; *X* = 46, *Y* = −70, *Z* = 16). The visualization of the relationship is shown in the scatterplot in the bottom right corner. Images are presented in the radiological convention. A-anterior, I-inferior, L-left, P-posterior, R-right, S-superior. IC, Independent Component.

Greater self-reported impulsivity (BIS score) was associated with lower functional connectivity of the right lateral occipital cortex with IC7, a network that correlated significantly with Somatomotor template network (peak mm 46/-70/16, FWE 1-*p* = 0.981) (Figure 2).

### Between-Network Connectivity

**FIGURE 3:**
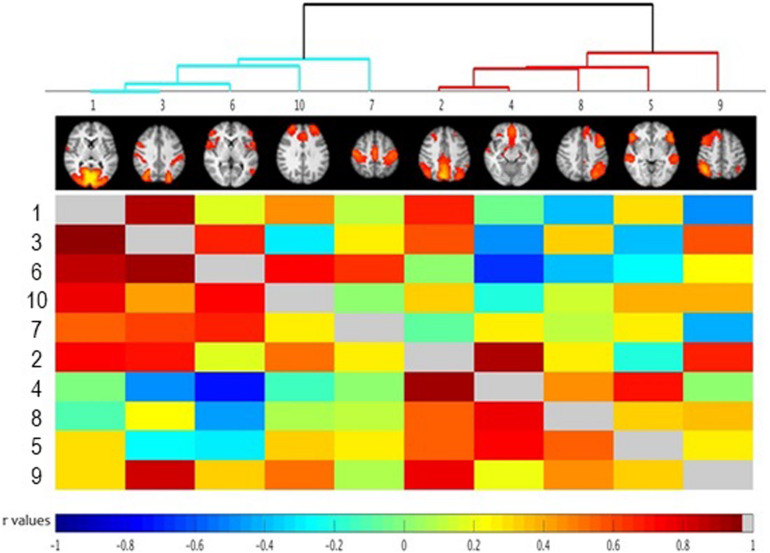
FSLNets results of between network correlations (*N* = 30). Each independent component (IC) is denoted by one column and a corresponding raw. The colored matrix displays the correlations of the time series between networks pairs. Dark red squares indicate highly positive correlations, light green indicates a near-0 correlation, and dark blue represents highly negative correlation as denoted on the scale at the bottom of the figure. Full correlations between networks pairs are shown below the diagonal line (in gray) with partial correlations shown above the diagonal line (for detailed description of full and partial correlation please see main text). Groups of highly correlated ICs were clustered together according to a hierarchical clustering algorithm (visualized at the top of the matrix as a clustering tree). Please note that the color cut-off for hierarchical tree is arbitrary – just for visualization purposes. Numbers indicate specific independent components as described in Table 1. The ICs have been reordered, according to a hierarchical clustering algorithm. Small images at the top of each column summarize each IC’s spatial map, with the right side of the images representing the left side of the brain. As described in the main text, the between-network connectivity was not modulated by trait impulsivity score.

Network analysis using FSLNets revealed a modular structure of functional networks, which could be segregated into clusters: Cluster 1 comprised of Visual, Somatomotor as well as Ventral and Dorsal Attention Networks (Figure 3, blue cluster), while Cluster 2 comprised of Frontoparietal and Default-Mode Networks (Figure 3, red cluster).

Using BIS as a predictor, no significant between-network differences in connectivity were found.

## Discussion

This study investigated whether aspects of intrinsic functional architecture and between-network connectivity pattern is associated with individual differences in trait impulsivity in a normative (university) population. We showed that individual differences in trait impulsivity, assessed with BIS Total Score, are associated with altered aspects of the functional architecture of the Somatomotor RS network. Specifically, higher trait impulsivity was linked to decreased coupling between the lateral occipital cortex and the Somatomotor Network. Surprisingly, we did not find any significant differences in the network functional architecture of default mode or frontoparietal networks associated with impulsivity, as has been reported previously (Inuggi et al., 2014; Reineberg et al., 2015). However, it is important to note that such previous research used different measures of impulsivity. Therefore, those inconsistent findings might merely reflect a heterogeneous nature of impulsivity and its underlying neural mechanisms (Caswell et al., 2015; Herman et al., 2018).

The finding of disrupted FC within the Somatomotor RS network in relation to trait impulsivity level corroborates previous studies. The graph theory approach, has been used to test the relationship between impulsivity (as reflected in BIS score) and the functional segregation (i.e., modularity) of whole-brain resting state architecture (Davis et al., 2013). Overall, this reveals a shift in the functional connectivity between visual, sensorimotor, cortical, and subcortical structures across the impulsivity range; specifically pointing to increased functional modularity between cortical and sub-cortical regions as a function of impulsivity score.

The lateral occipital cortex supports both visual perception and multisensory integration (Grill-Spector et al., 2001; Beauchamp, 2005). Interestingly, it is recognized that visual cortices contribute to impulsivity (Davis et al., 2013) and disorders commonly associated with impulsivity, including as Attention Deficit-Hyperactivity Disorder (ADHD; Castellanos and Proal, 2013). The sensorimotor network consists of both motor cortices, known to play a critical role in response inhibition (Li et al., 2006; Duque et al., 2012; Rae et al., 2014), and somatosensory areas, which are vital for sensory integration. These regions show altered activity in inhibitory control in diseased states such as post-traumatic stress disorder (Falconer et al., 2008; van Rooij et al., 2014) or under pharmacological interventions with LSD (Schmidt et al., 2017). Here, the “decoupling” may itself reflect a deficit in effective integration of perceptual information (visual and somatosensory cortices) with somatomotor outputs (motor cortex) associated with behavioral control, ultimately resulting in negative consequences, from poor planning for the future to excessive substance use (Dickman, 1990; Herman and Duka, 2019).

### Limitations

Some limitations merit comment. Our study was conducted on a moderately sized sample of students and employees of the university. The average BIS total score in our sample is 65.30 ± 11.39; which is consistent with other reports in the literature of university sample [e.g., 65.67 ± 9.92 in males and 64.58 ± 10.36 in females according to Caswell et al. (2015) and 63.82 ± 10.17 according to Patton et al. (1995)] and normative community populations [59.18 ± 9.54 according to Reise et al. (2013) or 62.3 ± 10.3 according to Stanford et al. (2009)]. However, our sample consists of relatively high-functioning young adults, who may have developed many mechanisms to cope with elevated impulsivity levels in daily life, which might have an effect on aspects of functional connectivity. It is also important to mention that the majority of the sample consisted of females, some of which were using hormonal contraception, which can affect functional connectivity (Hausmann, 2005). Therefore, future research should replicate our findings in larger-scale studies with general population, including a range of individuals with various backgrounds and educational levels. Finally, we did not find any suprathreshold differences in between-network connectivity that could be related to elevated impulsivity levels. Possibly, this is because our sample consisted of highly functioning young adults, all from the university population, and differences in between-network connectivity may only reveal themselves in pathologically impulsive individuals.

## Conclusion

In the brain, aspects of the functional architecture of the Somatomotor Network were associated with individual differences in trait impulsivity (BIS Total score). Specifically, more impulsive individuals showed decreased connectivity between the lateral occipital cortex and the Somatomotor Network. Since perception informs action and vice versa (Creem-Regehr and Kunz, 2010), proper integration of sensory inputs is crucial for adaptive behavioral responses. Therefore, the observed decreased connectivity between the visual and somatosensory cortices and motor cortex, may reflect itself in less effective integration of perceptual information and behavioral control and, thus, in negative consequences. However, in this normative sample, the between-network architecture was not related to trait impulsivity level. This evidence supports the use of RS FC-approaches to identify biomarkers for impulse-control problems.

## Data Availability Statement

The datasets generated for this study are available on request to the corresponding author. All behavioral data is available within the manuscript.

## Ethics Statement

The studies involving human participants were reviewed and approved by the Brighton and Sussex Medical School Research Governance and Ethics Committee. The patients/participants provided their written informed consent to participate in this study.

## Author Contributions

AH, HC, and TD were responsible for the study concept and design. AH carried out the study and the data analysis and drafted the manuscript. AH and TD interpreted the findings. HC and TD provided critical revisions of the manuscript for important intellectual content. All authors contributed to the article and approved the submitted version.

## Conflict of Interest

The authors declare that the research was conducted in the absence of any commercial or financial relationships that could be construed as a potential conflict of interest.

## Footnotes

1 http://fsl.fmrib.ox.ac.uk/fsl/fslwiki/FSLNets
